# Applicability of Redirecting Artemisinins for New Targets

**DOI:** 10.1002/gch2.202300030

**Published:** 2023-10-27

**Authors:** Jacob Golenser, Nicholas H. Hunt, Ida Birman, Charles L. Jaffe, Johanna Zech, Karsten Mäder, Daniel Gold

**Affiliations:** ^1^ Department of Microbiology and Molecular Genetics Kuvin Center for the Study of Infectious and Tropical Diseases The Hebrew University – Hadassah Medical Center Jerusalem Israel; ^2^ School of Medical Sciences University of Sydney Sydney 2050 Australia; ^3^ Institute of Pharmacy Martin Luther University Halle‐Wittenberg 06108 Halle Germany; ^4^ Department of Clinical Microbiology and Immunology Faculty of Medicine Tel Aviv University Tel Aviv Israel

**Keywords:** bacterial infections, cancer, microbiota, parasites, repurposing artemisinin, viral infections

## Abstract

Employing new therapeutic indications for drugs that are already approved for human use has obvious advantages, including reduced costs and timelines, because some routine steps of drug development and regulation are not required. This work concentrates on the redirection of artemisinins (ARTS) that already are approved for clinical use, or investigated, for malaria treatment. Several mechanisms of action are suggested for ARTS, among which only a few have been successfully examined in vivo, mainly the induction of oxidant stress and anti‐inflammatory effects. Despite these seemingly contradictory effects, ARTS are proposed for repurposing in treatment of inflammatory disorders and diverse types of diseases caused by viral, bacterial, fungal, and parasitic infections. When pathogens are treated the expected outcome is diminution of the causative agents and/or their inflammatory damage. In general, repurposing ARTS is successful in only a very few cases, specifically when a valid mechanism can be targeted using an additional therapeutic agent and appropriate drug delivery. Investigation of repurposing should include optimization of drug combinations followed by examination in relevant cell lines, organoids, and animal models, before moving to clinical trials.

## Graphical abstract

Repurposing of drugs has great practical appeal. However, a systematic approach is essential. Here, artemisinins (ARTS) are used to illustrate some considerations that are critical in this process. Several mechanisms of action are suggested for ARTS, among which only a few are successfully examined in vivo, mainly the induction of oxidant stress and anti‐inflammatory effects. Consequently, ARTS are proposed for repurposing in treatment of inflammatory disorders and diverse types of diseases caused by viral, bacterial, fungal, and parasitic infections. In general, repurposing ARTS is successful in only a very few cases, specifically when a valid mechanism can be targeted, with the use of an additional therapeutic agent and appropriate drug delivery.

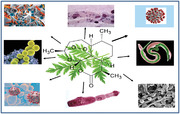

## 1 Introduction

Drug repurposing/redirecting, a strategy to identify new uses for approved or investigational drugs outside the scope of the original medical indication, offers various advantages over developing an entirely new drug for a given indication.^[^ ^1^ ^]^ However, despite these advantages, there are still major technological and regulatory challenges that need to be addressed.^[^ ^2^ ^]^ Recently, because of the SARS‐COVID‐19 outbreak, a trend emerged to utilize drugs currently employed against malaria and filariasis, e.g., ivermectin, chloroquine, and artemisinins (ARTS), for the treatment of this viral disease. Irrespective of their original use, these drugs have been proposed as treatments for a variety of diseases. In the current article, we aim to exemplify the wider scope of drug repurposing by considering ARTS. We describe the alleged justification for ARTS usage based on their likely mechanisms of action, the variety of diseases tested, and consider recent failures and achievements of repurposing supported by actual experimental examples. Overall, we emphasize consideration of the applicability of ARTS repurposing.

**Figure 1:**
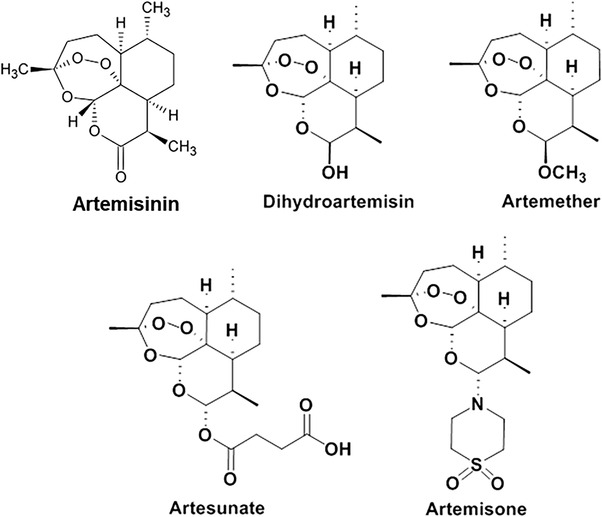
Structures of artemisinin and some of its derivatives.

Artemisinin was isolated by Youyou in 1972 for malaria treatment.^[^ ^3^ ^]^ It is a sesquiterpene lactone that includes a peroxide bridge, which is responsible for its activity (**Figure** **1**). Following the global emergence of chloroquine resistance,^[^ ^4^ ^]^ ARTS have become the first‐line treatment of malaria. A few ARTS have been approved in this respect, namely dihydroartemisinin (DHA), artesunate, and artemether, but only when used in artemisinin‐based combinations (ACT) with other drugs.^[^ ^5^ ^]^ Many non‐approved ARTS that originally were suggested as potential antimalarials also have been proposed for other medical purposes.

Various mechanisms have been suggested for the activity of ARTS.^[^ ^6^, ^7^, ^8^ ^]^ For the most part, these reviews deal with the mode of activation of ARTS and their intracellular targets. Meshnick et al.,^[^ ^9^ ^]^ suggested radical effects following triggering of artemisinin by ring opening mediated by heme and Fe^2+^. However, ARTS susceptible to decomposition by heme‐Fe^2+^ display enhanced activities against parasites cultured under carbon monoxide (CO), an agent that passivates heme‐Fe^2+^ by formation of stable heme‐Fe^2+^‐CO complexes; this thereby discounts heme as an activator of ARTS.^[^ ^10^, ^11^, ^12^ ^]^ According to this concept, ARTS may also act via other pathways, e.g. through oxidation of FADH2.^[^ ^13^, ^14^ ^]^ The common dogma is that both iron‐dependent and ‐independent reaction pathways of ARTS are related to perturbation of redox homeostasis that ultimately leads to generation of reactive oxygen species (ROS). Nevertheless, ARTS can act independently of oxidative damage by interfering with iron transport via transferrin receptor‐1 in a non‐classical endocytic pathway.^[^ ^14^ ^]^

The most common artemisinin effects are presented in **Table** **1**. Some of the reported treatment effects appear to be contradictory, e.g. anti‐inflammatory effects versus oxidant stress. Others might be interconnected, such as effects on P‐glycoprotein and membrane permeability, cell cycle arrest, induction of apoptosis,^[^ ^8^ ^]^ and interference with the activity of ion channels.^[^ ^35^ ^]^ However, the most relevant mechanisms that have been confirmed in vivo are the induction of oxidant stress and the anti‐inflammatory effects. Likewise, free radicals may cause protein damage and inhibit folding of newly synthesized proteins. This likely leads to perturbation of cell function and viability.^[^ ^9^, ^16^, ^17^ ^]^ In addition, ARTS may affect divergent immune responses that are related to inflammation and autoimmunity, e.g. in ocular fibrosis,^[^ ^36^ ^]^ rheumatoid arthritis, lupus erythematosus, and various allergic diseases.^[^ ^19^ ^]^ Both activities, i.e., those associated with parasite elimination and immunomodulation, are vital for avoiding severe malaria, especially cerebral malaria (CM) and the respiratory distress syndrome that are induced by *Plasmodium falciparum*.

**Table 1:** Suggested mechanisms of action of artemisinins.

Mechanism	Reference
^*^ Perturbation of redox homeostasis associated with oxidant stress a. related to iron intervention. b. iron‐independent	[9, 15, 16, 17, 18] [10, 11, 12, 13]
^*^ Anti‐inflammatory activity.	[19, 20, 21]
^*^ Induction of apoptosis.	[22, 23, 24]
^*^ Ferroptosis.	[25, 26, 27]
Interference with Vascular Endothelial Growth Factor (VEGF) activity.	[28]
Overcoming P‐glycoprotein‐mediated multi‐drug resistance.	[29]
Changing membrane permeability	[30]
Interference with plasmodia sarcoplasmic/endoplasmic reticulum Ca++ ATPase (SERCA).	[31]
Targeting parasite mitochondria.	[32]
^*^ Regulating the expression levels of metastatic tumor antigens.	[33]
^*^ Suppressing lung cancer cells by down‐regulating the AKT/Survivin signaling pathway.	[34]

^*^ Confirmed in vivo in some of the quoted references.

The proposed mechanisms of ARTS activity have initiated a flood of suggested uses for ARTS in treating diverse types of diseases, including viral, bacterial, fungal, and parasitic infections;^[^ ^18^, ^37^, ^38^, ^39^ ^]^ inflammatory diseases;^[^ ^19^ ^]^ autoimmune diseases;^[^ ^40^ ^]^ cancer;^[^ ^19^, ^41^ ^]^ and ischemic brain damage.^[^ ^42^, ^43^, ^44^ ^]^ Many of the suggested uses are based on molecular drug/target docking or theoretical computational analyses, or on preliminary in vitro experiments that employ high ARTS concentrations. The equivalent amounts of drug, if used in vivo, would likely worsen rather than ameliorate the diseases under study, due to their side effects.

In this concise review, examples of suggested ARTS repurposing are presented and critically evaluated.

## 2 Redirecting ARTS

### 2.1 Cytomegalovirus (CMV)

ARTS treatment of malaria during pregnancy has been debated, due to alleged embryotoxicity.^[^ ^45^ ^]^ Recently, the WHO has recommended that artesunate in combination with other drugs may be used to treat severe malaria during pregnancy.^[^ ^5^ ^]^ In view of the spreading plasmodial resistance to ARTS, artemisone, a 10‐alkylo artemisinin (Figure 1), was considered as an improved new artemisinin derivative for malaria therapy^[^ ^46^, ^47^ ^]^ and later for treatment of CMV.^[^ ^48^ ^]^ This idea was based on the results of experiments in CMV cultures that demonstrated an EC50 of ≈1 µM, about 900‐fold the EC50 of artemisone in *Plasmodium falciparum* culture. Employment of an additional anti‐viral inhibitor of CMV reduced the artemisone concentration in the combination.^[^ ^49^ ^]^ CMV causes devastating disease during pregnancy.^[^ ^50^ ^]^ Unfortunately, in vivo treatment of CMV infection with corresponding amounts of artemisone, considering the recommended treatment for malaria using ARTS, would likely be an overdose, especially during pregnancy.^[^ ^45^ ^]^ It is noteworthy that both the efficacy and toxicity of artemisone are increased, in comparison to other ARTS, because of the lack of first‐pass metabolism of this drug.^[^ ^46^ ^]^ A similar problem is relevant for artesunate (**Table** **2**). A slow‐release system for the drugs might mitigate the potential toxicity. In a clinical trial, CMV was not affected by artesunate.^[^ ^51^ ^]^ Some field studies concerning the efficiency of ARTS against CMV were conducted in malaria patients. Artemether‐lumefantrine decreased urine viral load, but artemether alone was not examined and the effects of malaria per se on viral load were not evaluated.^[^ ^52^ ^]^ Overall, attempts to treat patients with ARTS alone, or ARTS in combination with other antimalarials or with anti‐viral drugs, do not provide clear evidence for the efficacy of ARTS against CMV.^[^ ^51^ ^]^

**Table 2:** In vitro effects of artemisinins against *Plasmodium falciparum* and *Cytomegalovirus*.

Pathogen	ED^[^ ^50^ ^]^	Reference
Artemisone vs. *P. falciparum*	1.1^±^0.6 nM	[53]
^*^ Artemisone vs *Cytomegalovirus*	1000^±^300 nM	[48]
Artesunate vs *P. falciparum*	11.2^±^3.8 nM	[54]
Artesunate vs Cy*tomegalovirus*	5800^±^400 nM	[55]

^*^ Compared with a combination of Artemisone + Maribavir.

VS. *Cytomegalovirus*, 480^±^260 nM.^[^ ^49^ ^]^

### 2.2 COVID‐19

**Figure 2:**
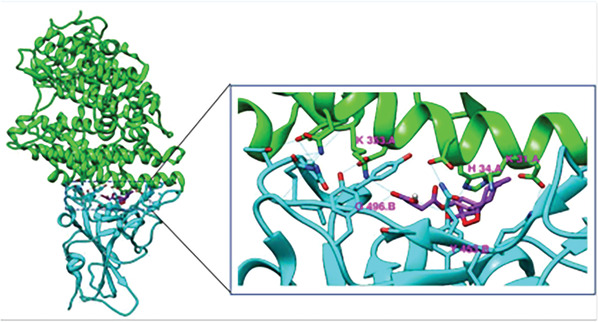
Artesunate alignment in its binding to SARS‐CoV‐2 spike protein. Artesunate aligned in the binding interface of the S‐protein (viral) RBD‐hACE2 complex. Shown in cyan is the S‐Protein RBD and in green hACE2 (the human receptor). Artesunate is shown in purple^[^ ^63^ ^]^ (permission allocated).

Currently, a limited number of drugs are approved for treatment of COVID‐19,^[^ ^56^ ^]^ namely, the anti‐protease PAXLOVID^[^ ^57^ ^]^ and the nucleoside analogs Molnupiravir and Remdesivi^[^ ^58^ ^]^ The pathology of this viral disease has some similarities to that of malarial acute respiratory distress syndrome, in that the lungs are the main target organ, and the injury and death are immune‐mediated, rather than pathogen‐mediated.^[^ ^59^, ^60^ ^]^ Consequently, ARTS treatment against COVID‐19 has been proposed, based on its anti‐inflammatory activity,^[^ ^8^, ^9^, ^38^, ^61^ ^]^ inactivation of viral papain‐like protease^[^ ^62^ ^]^ and also the specific binding of artesunate to the spike protein of SARS‐CoV‐2 (**Figure** **2**, docking).

So far, few experiments have been conducted to assess the effect of ARTS on COVID‐19. Most of these were tested in vitro or by in silico docking on SARS‐CoV‐2. Arteannuin B showed the highest inhibition, with an EC50 of 10.3 ± 1.1 µM, but the CC50 was 71.1 ± 2.5 µM,^[^ ^64^ ^]^ which suggests a likely narrow therapeutic index. In a clinical study, artemisinin combined with piperaquine (AP) was reported to have a significant impact on COVID‐19. The study included 23 treated and 18 control individuals: per *os* tablets composed of 62.5 mg artemisinin and 375 mg piperaquine were compared with 400 mg hydroxychloroquine and 400 mg Arbidol throughout a seven‐day treatment.^[^ ^65^ ^]^ AP significantly shortened from 19 to 11 days the time to reach undetectable SARS‐CoV‐2 (**Table** **3**). Previously, AP therapy in malaria patients was found, in some cases, to cause QT interval prolongation in electrocardiograms.^[^ ^66^ ^]^ These results should be considered when treating COVID‐19 with AP. In general, there is no convincing proof of the clinical benefit of ARTS against the manifestations of infection with SARS‐CoV‐2.

**Table 3:** The effect of artemisinins on COVID‐19.

Overview based on 19 studies^[^ ^38^ ^]^ 4 in vitro 14 in silico 1 clinical trial ↓ A clinical trial in mild to moderate illness^[^ ^65^ ^]^ Artemisinin+Piperaquine Vs. Hydroxychloroquine+Arbidol (Umifenovir): Artemisinin+piperaquine treatment significantly shortened the time to reach undetectable SARS‐CoV‐2 from 19 to 11 days

Overall, attempts at repurposing drugs (approved for human administration) or redirecting (not yet approved) against COVID‐19 have not been fruitful, possibly because of the small number of compounds tested using classical experimental approaches and the reliance on a limited set of factors that were used to predict the relevance of the drugs that were being considered.^[^ ^67^ ^]^ To try to overcome these deficiencies, Ribaudo and colleagues^[^ ^61^ ^]^ used computational methods to examine anti‐malarial drugs that may target the papain‐like protease (PLpro) of SARS‐CoV‐2. ARTS and amodiaquine were selected for further analysis by binding site profiling, induced‐fit docking, and molecular dynamics. These predictions were examined using a SARS‐CoV‐2 PLpro assay kit measuring deubiquitinase activity and the results were that amodiaquine may be a potential anti‐viral drug but that ARTS does not qualify. Fiscon and Paci^[^ ^68^ ^]^ suggest a computational network‐based approach to examining marketed drugs for their potential repurposing for COVID‐19 treatment. It should be “a network‐based algorithm taking as input a list of drug targets and disease genes, predicting drug‐disease associations by computing a new network‐based similarity measure to prioritize associatioennyns between drugs and diseases located in the same network neighborhoods”. Using this approach, Fiscon and Paci^[^ ^67^ ^]^ found 98 network‐predicted drugs with the potential to treat SARS‐CoV‐2 infection. Gysi et al,^[^ ^67^ ^]^ employed algorithms relying on artificial intelligence, network diffusion, and network proximity. They examined 918 drugs that previously had been screened for toxicity and were considered as potential compounds for combating COVID‐19. It is noteworthy that there was no single predictive algorithm that offered a reliable outcome: none of the compounds eventually reduced viral infection‐bound proteins that theoretically might be targeted by SARS‐COV‐2. Apparently, the inhibition of viral development might be mediated by changes inflicted by the drugs on the host cells. Consequently, the authors concluded that predictive methods should not rely on a single parameter and that docking should be only one of the predictive strategies. Gysi et al.,^[^ ^67^ ^]^ ranked 6340 compounds associated with anti‐viral activity, but ARTS were not among the first 100 compounds with positive outcomes.

### 2.3 Bacterial Infection

ARTS efficacy against bacterial pathogens has been examined in vitro; for example, their anti‐mycobacterial activity was inferior by two orders of magnitudes to that of Rifampicin.^[^ ^69^ ^]^ Other in vitro trials revealed a potential use of artemisinin against *Helicobacter pylori*, both when combined with gentamycin^[^ ^70^ ^]^ or alone.^[^ ^71^ ^]^ Lin et al.,^[^ ^15^ ^]^ reported an in vitro anti‐*Staphylococcus aureus* effect of artemisinin complexed in beta‐cyclodextrin: 20 mg mL^−1^ abolished the bacteria within four days. They suggested a new mechanism of artemisinin, by inducing increased membrane permeability. However, this could be a secondary effect following the destruction of macromolecules by free radicals; in addition, the artemisinin concentration was enormous, and effects on control eukaryotic cells were not evaluated.

### 2.4 Microbiota

An interrelationship between ARTS, the microbiome, and various diseases has been suggested as a new angle for drug development. Unfortunately, based on the current scientific literature (i.e., “The first Parasite Microbiome Project Workshop”,^[^ ^72^, ^73^ ^]^ a definitive conclusion on the role of ARTS therapy in this respect cannot yet be drawn. Changes in the microbiome, either through diet^[^ ^74^ ^]^ or drugs, may affect various maladies, e.g. parasitic^[^ ^75^ ^]^ or rheumatic^[^ ^76^ ^]^ diseases. Moreover, certain bacteria may induce immunomodulation that in turn could ameliorate severe diseases.^[^ ^77^ ^]^ Thus, for example, changes in the composition of the gut microbiota affect the severity of malaria.^[^ ^78^, ^79^ ^]^ Treatment of mice with DHA induced significant changes in the gut microbiome.^[^ ^80^ ^]^ Furthermore, DHA ameliorated the inflammatory bowel disease induced by dextran sulfate sodium in mice, and this positive effect coincided with changes in the gut microbiota.^[^ ^81^ ^]^ These results are in line with the hypothesis that the composition of the gut microbiota modulates the strictness of malaria.^[^ ^82^ ^]^ In contrast, Denny and Schmidt^[^ ^82^ ^]^ could not show any effect of ACT on the gut microbiota of mice, and no change was found in the bacteria communities of infant stool, before and after acute febrile malaria and artemether‐lumefantrine treatment.^[^ ^83^ ^]^

### 2.5 Parasites

Some stages of the generally complicated lifecycles of parasites are easy to maintain in vitro and, consequently, initial drug testing is usually conducted in that way. Unfortunately, in vivo experiments may not duplicate the anti‐parasitic effect demonstrated in vitro. For instance, anti‐histomonal effects in vitro of artemisinin and *Artemisia annua* extracts could not be confirmed in turkeys and chickens infected with *Histomonas meleagridis*.^[^ ^84^ ^]^

New artemisinin derivatives have been tested in vitro against *Toxoplasma gondii*. The methylesther and 2‐thiazole derivatives were most active against the tachyzoites and their cell invasion and had minimal cytotoxicity. These C‐10‐carba‐linked derivatives are designed to be much less susceptible to P450 oxidation, with the intention of minimizing possible neurotoxicity due to DHA.^[^ ^85^ ^]^ However, this speculation was not verified. Rosenberg and colleagues,^[^ ^86^ ^]^ by genome editing using CRISPR/Cas9, induced artemisinin resistance of *T. gondii* that was associated with mitochondrial function. Resistance to artemisinin and DHA previously had been induced by exposing toxoplasma cultures to increasing concentrations of the ARTS.^[^ ^87^ ^]^

**Figure 3:**
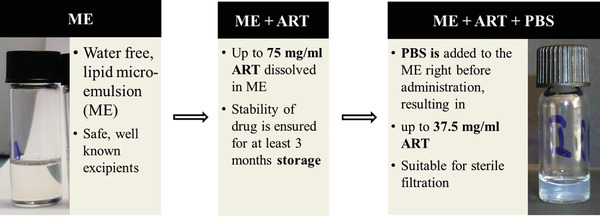
Formulation of artemisone (ART) in lipid micro‐emulsion. Artemisone (ART) shows poor water solubility (89 ng mL^−1^) and decomposition in aqueous environment (pH‐dependent). The solubility of the drug is greatly increased (∼ ×1000) in the lipid excipients.

**Figure 4:**
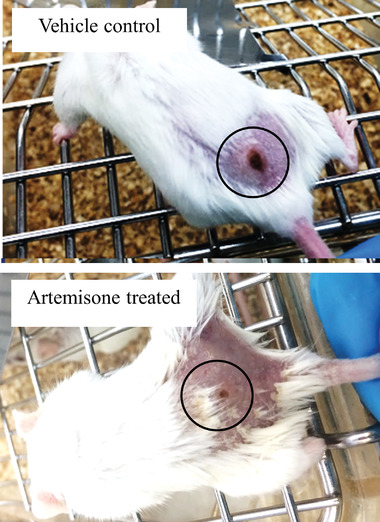
Treatment of cutaneous leishmaniasis by artemisone. BALB/c mice were infected subcutaneously on the lower back just above the tail with *Leishmania major* Friedlin strain (5 × 10^6^ metacyclic promastigotes) as previously described.^[^ ^95^ ^]^ Lesions appeared on day 7. Mice were treated twice a day on days 8–11 and 14–17 post‐infection (PI) by spraying artemisone (1 mg per dose). Representative lesions for the control (*n* = 5) and treated (*n* = 6) mice on day 25 PI are shown. The average lesion size was (mean±SE) 51.5^±^6.6 and 28.1^±^3.2 mm^2^ for each group, respectively (*p<*0.05, Prisme paired T‐test), (Golenser et al. unpublished results). The spraying method is detailed in Zech et al.,^[^ ^90^ ^]^ This research conforms to the Hebrew University Animal Ethical Committee guidelines, ethics number MD‐14‐13923‐3.

Aucamp et al.,^[^ ^88^ ^]^ found that their newly synthesized ARTS were as much as 30‐fold more potent against *Leishmania* promastigotes than the currently used commercial ARTS. However, the promastigote stage is not the one responsible for clinical leishmaniasis in humans or animals; drugs that act against a certain stage in the parasite lifecycle are not necessarily active against another stage. Therefore, it is crucial in anti‐leishmanial drug screening to test against the intracellular amastigote, which is responsible for the disease pathology. Testing of ARTS against leishmaniasis in animal models yielded unsatisfactory outcomes. Zech and co‐workers developed a new microemulsion for delivering high quantities of ARTS in vivo^[^ ^89^ ^]^ (**Figure** **3**). Following successful transdermal treatment of murine malaria by artemisone incorporated in the microemulsion,^[^ ^90^ ^]^ the activity of the formulation against cutaneous leishmaniasis was examined in a BALB/c mouse model (Golenser et al. unpublished results) and there was a significant reduction in lesion size (**Figure** **4**). However, the treated mice eventually became moribund a few weeks after the control ones. Interestingly, nano‐liposomal artesunate significantly reduced parasite load in the livers and spleens of mice with visceral leishmaniasis^[^ ^91^ ^]^ Whilst free radical production occurs in blood‐dwelling malaria parasites, due to the availability of labile iron, this mechanism of ARTS killing might not be valid in established cutaneous leishmaniasis, where labile iron may not be available. However, none of a series of non‐peroxide ARTS tested had leishmanicidal activity,^[^ ^92^ ^]^ showing that free radicals are involved in affecting leishmanial development. Both *Leishmania* promastigotes and amastigotes developed ARTS resistance under drug pressure, perhaps explaining the above‐mentioned in vivo drug failure.^[^ ^93^ ^]^ It is possible that a different mode of delivery,^[^ ^91^ ^]^ or ARTS combined with a second anti‐leishmanial drug, would prevent resistance and thus could be used as an alternative to the current treatment protocols. This assumption is supported by the efficacy of DHA together with amphotericin B against another disease, candidiasis, in a murine model. DHA alone did not affect the disease but elevated the ergosterol levels of the *Candida*.^[^ ^94^ ^]^ Thus, the synergistic effect is likely expressed because *Candida* (like leishmania parasites) possesses ergosterol – which is specifically sensitive to amphotericin B – as its main membrane lipid.

**Figure 5:**
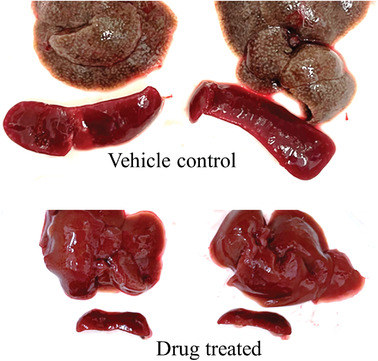
Gross macroscopic observation of livers of *Schistosoma*‐infected mice, vehicle or artemisone‐treated. Representative photographs are shown of liver lobes and spleens from male mice infected with schistosomes and then treated with either a placebo (*n* = 5, upper photographs) or artemisone (*n* = 5, lower photographs). Mice were treated by gavage at 23–25‐ and 29–31 days post‐infection twice a day, with 40 mg k^−1^g, in microemulsion. Worm number in the control and drug‐treated mice was 48^±^16 and 1.5^±^0.4 (*p<*0.01 by Prism T‐test), respectively. The spleen weights of the control mice were about 20 times higher than that of the treated ones. Formulation and treatment methods are detailed elsewhere.^[^ ^89^ ^]^

The effects of DHA on *Candida* should be interpreted in view of mechanisms specific to that species of pathogen, as should the inhibition of *Schistosoma* development by ARTS. Because both malaria and schistosomiasis are caused by blood‐dwelling parasites, it was speculated that ARTS are active against the schistosomes via heme‐initiated formation of free radicals. Therefore, various ARTS have been suggested as an alternative to praziquantel treatment of schistosomiasis.^[^ ^89^, ^96^, ^97^, ^98^, ^99^ ^]^ A successful outcome using oral delivery of artemisone is demonstrated in **Figure** **5**. Worm number was profoundly reduced and, consequently, granulomas were not observed and the spleen sizes were similar to those of untreated normal mice.^[^ ^89^ ^]^

Contradictory results have been reported concerning the treatment of another worm, *Echinococcus granulosus*, with artesunate: whilst Wen et al.,^[^ ^100^ ^]^ showed in vivo and in vitro antiparasitic effects, significant results were obtained only in vitro following exposure to DHA and artesunate and the severity of echinococcosis in mice was not affected by the treatment.^[^ ^101^ ^]^

### 2.6 Cancer

A search of PubMed (September 12, 2023) reveals 1248 publications on ARTS for treatment of cancer; most of these describe the successful in vitro treatment of cell lines. However, a few successful experiments were recently performed in mouse models and are briefly discussed here. Likewise, Jia et al.,^[^ ^102^ ^]^ found that artesunate ameliorates irinotecan‐induced intestinal injury by suppressing cellular senescence and significantly enhances anti‐tumor activity, Zaho C. et al.,^[^ ^33^ ^]^ reported that artesunate may affect metastatic tumor by regulating the expression levels of the tumor's antigens and Zhang W. et al.,^[^ ^34^ ^]^ demonstrated suppression of lung cancer cells by downregulating the AKT/Survivin signaling pathway. Peng and colleagues^[^ ^103^ ^]^ found that targeted lipid nanoparticles encapsulating DHA and chloroquine phosphate suppressed the proliferation and liver metastasis of colorectal cancer. 2‐Carbon‐linked dimeric artemisinin‐derived analogs together with anti‐neoplastic drugs had an additive therapeutic effect on acute myeloid leukemia in immunodeficient mice.^[^ ^104^ ^]^ Li et al.,^[^ ^105^ ^]^ demonstrated a new nano‐system for releasing artemisinin that enhanced ferroptosis and breast tumor inhibition in nude mice. While the effects in immune‐compromised mice might be compared to drug effects in immune‐deficient human individuals, results in intact subjects might be different, depending on any interaction between the drugs and the relevant immune responses. Another delivery system, a biodegradable poly(ethylene glycol) methyl ether‐poly(ε‐caprolactone) micelle carrier for DHA, showed higher therapeutic efficacy and lower toxicity than the free drug in vivo and significantly inhibited cervical tumor growth in mice.^[^ ^106^ ^]^

The publications cited above imply that ARTS have potential as anti‐cancer therapeutics. However, only a few preliminary clinical trials have been carried out in which ARTS treatment was combined with conventional chemotherapy, and information on the effect on patient survival is lacking. Even though judicious use of new delivery modes, drug combinations, and targeting might well increase the potential value of ARTS therapy, experiments in mouse models don't necessarily predict the outcomes in human cancer treatment.

## 3 Conclusions

ARTS have been synthesized for malaria therapy and, later, suggested for other clinical applications.^[^ ^19^ ^]^ In the current analysis, we have concentrated on the applicability of ARTS for treatment of other diseases. Currently, ARTS are administered in malaria therapy by injection, transfusion, oral delivery, intranasal dispatch, and the intrarectal route.^[^ ^107^ ^]^ Furthermore, there is abundant recent research aimed at developing new delivery systems for ARTS. Using these techniques, it is also possible to target specific organs or cells, for example, the brain in order to specifically treat CM,^[^ ^108^ ^]^ or magnetically‐targeted drug delivery systems composed of nanoparticles co‐loaded with artemisinin that specifically target hemozoin, a vital metabolite of *Plasmodium* in its erythrocytic stage.^[^ ^109^ ^]^ In general, drug targeting should be optimized for the disease in question, the affected organ, and the unique metabolism of the pathogen.

However, refined targeting is not sufficient for predicting the success of novel ARTS, or indeed any medication. For example, any suggestion of repurposing or redirecting existing compounds should consider and test the risk of the causative pathogen acquiring resistance. This has not been accomplished in most of the published studies that have considered an altered use of the drugs in question. In general, ARTS are not yet applicable for treatment of diseases other than malaria, except for possible use against schistosomes, where it might be suitable because of the common metabolism associated with ingestion of hemoglobin by the pathogen.^[^ ^89^ ^]^

**Figure 6:**
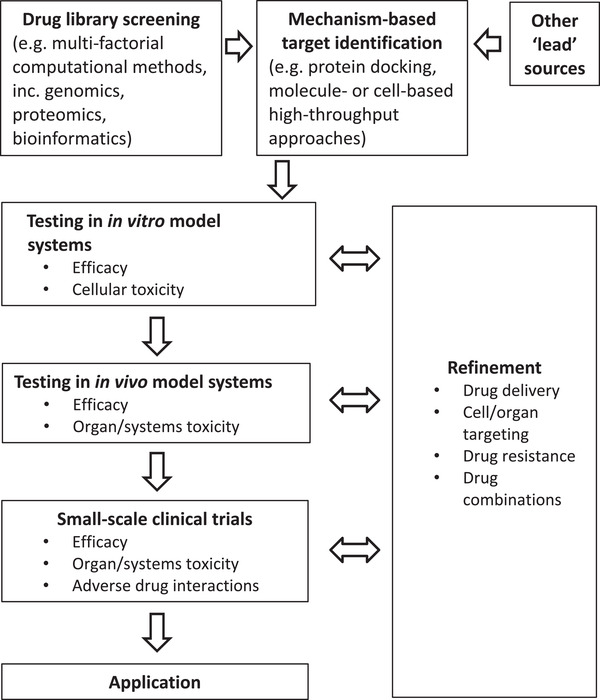
Targeting toward applicability.

Future experiments aiming at new ARTS uses should include at least one additional drug^[^ ^12^ ^]^ and in vivo validation in relevant models^[^ ^110^ ^]^ before a definitive conclusion is drawn concerning applicability. One pertinent example is the development of anti‐plasmodial drugs: these drugs are active in both human and mouse models.^[^ ^111^ ^]^ Methods based on ARTS screening via computational approaches, including proteomics, transcriptomics, and bioinformatics strategies to identify drug candidates, should be considered.^[^ ^61^ ^]^ From there, advanced docking techniques and high‐throughput selection,^[^ ^2^ ^]^ targeting a specific pathogen and its metabolism, as well as the affected organs, may result in improved outcomes (**Figure** **6**). The COVID pandemic provided examples of the unfortunate consequences of repurposing drugs without sufficient attention to systematic evaluation of this kind – for example, the controversial use of hydroxychloroquine.^[^ ^112^ ^]^

## Conflict of Interest

The authors declare no conflict of interest.
